# The Crystal Structures of Apo and cAMP-Bound GlxR from *Corynebacterium glutamicum* Reveal Structural and Dynamic Changes upon cAMP Binding in CRP/FNR Family Transcription Factors

**DOI:** 10.1371/journal.pone.0113265

**Published:** 2014-12-03

**Authors:** Philip D. Townsend, Britta Jungwirth, Florence Pojer, Michael Bußmann, Victoria A. Money, Stewart T. Cole, Alfred Pühler, Andreas Tauch, Michael Bott, Martin J. Cann, Ehmke Pohl

**Affiliations:** 1 School of Biological and Biomedical Sciences & Department of Chemistry, Biophysical Sciences Institute, Durham University, Durham, United Kingdom; 2 Institute for Genome Research and Systems Biology, Center for Biotechnology (CeBiTec), Bielefeld University, Bielefeld, Germany; 3 Global Health Institute, Protein Crystallography Core Facility, Ecole Poytechnique Fédérale de Lausanne (EPFL), Lausanne, Switzerland; 4 Institute of Bio- and Geosciences, IBG-1:Biotechnology, Forschungszentrum Jülich, Jülich, Germany; University of Washington, United States of America

## Abstract

The cyclic AMP-dependent transcriptional regulator GlxR from *Corynebacterium glutamicum* is a member of the super-family of CRP/FNR (cyclic AMP receptor protein/fumarate and nitrate reduction regulator) transcriptional regulators that play central roles in bacterial metabolic regulatory networks. In *C. glutamicum*, which is widely used for the industrial production of amino acids and serves as a non-pathogenic model organism for members of the *Corynebacteriales* including *Mycobacterium tuberculosis*, the GlxR homodimer controls the transcription of a large number of genes involved in carbon metabolism. GlxR therefore represents a key target for understanding the regulation and coordination of *C. glutamicum* metabolism. Here we investigate cylic AMP and DNA binding of GlxR from *C. glutamicum* and describe the crystal structures of apo GlxR determined at a resolution of 2.5 Å, and two crystal forms of holo GlxR at resolutions of 2.38 and 1.82 Å, respectively. The detailed structural analysis and comparison of GlxR with CRP reveals that the protein undergoes a distinctive conformational change upon cyclic AMP binding leading to a dimer structure more compatible to DNA-binding. As the two binding sites in the GlxR homodimer are structurally identical dynamic changes upon binding of the first ligand are responsible for the allosteric behavior. The results presented here show how dynamic and structural changes in GlxR lead to optimization of orientation and distance of its two DNA-binding helices for optimal DNA recognition.

## Introduction

The Gram-positive soil bacterium *Corynebacterium glutamicum* is widely used for the industrial production of amino-acids with an annual production of more than 4 Mt of L-glutamate and L-lysine combined [1]. Due to the rapidly increasing knowledge of *C. glutamicum* metabolism and regulation, numerous efforts are currently underway to use this bacterium for the production of an increasing range of small organic compounds including amino acids [2], [3], several organic acids such as succinate and lactate [4]–[6], but also the biofuels ethanol and isobutanol [7]–[9]. Many of these compounds are derived from intermediates of the tricarboxylic acid (TCA) cycle which is tightly regulated [10] on a transcriptional level by a range of DNA-binding proteins including the TetR-type AcnR [11], the LuxR-type RamA [12], the AraC-type RipA [13], as well as orthologues of the iron-dependent DtxR family [14] and most notably the CRP orthologue GlxR [15]. GlxR from *C. glutamicum* (Cg-GlxR) has been identified as one of the central regulators of the Corynebacterium metabolism and more than 200 genes under GlxR control have so far been identified [16]–[18]. Cg-GlxR, which shares a sequence identity of approximately 29% with CRP from *E. coli* (Ec-CRP), is a protein of 227 amino acids (compared to 210 for Ec-CRP) comprising an N-terminal dimerisation and ligand binding domain, and a C-terminal DNA-binding domain. The protein forms a homodimer with two structurally identical binding sites for cAMP. Once activated by cAMP, the regulator interacts with its target pseudo-palindromic DNA sites with the consensus sequence 5-TGTGANNTANNTCACA-3′ located in the regulatory region of Cg-GlxR controlled genes [16], [19]. In most cases Cg-GlxR functions as an activator where RNA polymerase is recruited by protein-protein-interactions thus enabling transcription of the downstream genes. In *E. coli* the intracellular level of cAMP has been shown to increase in response to glucose starvation via adenylate cyclase activation which in turn is linked to the bacterial phosphotransferase system (PTS) for sugar uptake [20]. In contrast, cAMP levels in *C. glutamicum* have been found to be decreased on acetate growth and increased during growth on glucose [15]. Cg-GlxR presents a central control point in the bacterial response to different nutrient sources and increasing our understanding of the molecular basis of activation and allostery may help to improve the regulation of metabolic processes.

Ec-CRP has for many years been the standard model to investigate allosteric cAMP binding and the molecular mechanism of DNA recognition. Although CRP was the first transcriptional regulator whose three-dimensional structure was determined by crystallography [21], and a number of structures of the regulator bound to cAMP and DNA have been determined by crystallography [22]–[24], the mechanism for how ligand binding causes activation of DNA binding remains controversial. The NMR structure suggested that cAMP binding resulted in a large movement of the DNA-binding domain coupled with a coil-to-helix transition extending into the coiled-coil region of the dimerisation interface [25]. In contrast, the medium resolution structure of apo Ec-CRP revealed a large albeit different hinge motion of the DNA-binding domains with respect to the dimerisation domain [26]. More recently, the crystal structure of apo CRP of *Mycobacterium tuberculosis* (Mt-CRP), which shares approximately 79% sequence identity with Cg-GlxR, showed an astonishing asymmetry between the protein monomers in one crystal structure, which was interpreted as a significant contributor to the activation mechanism [27]. However, no significant asymmetry was detected in solution for *E. coli* CRP by NMR [28] and it thus remains unclear if this is a general feature of activation in the CRP/FNR family. Whereas the first binding event of cAMP to CRP appears to involve structural changes in all cases investigated, the binding of the second cAMP in the structurally identical site of the second monomer is not accompanied by significant structural changes. Therefore, the remarkable negative allostery has been explained by changes in the dynamic behavior of the protein rather than defined conformational changes. The proposed dynamic changes have been supported by a number of elegant NMR experiments [25], [29], [30] as well as coarse-grain modeling studies [31]–[33]. In order to shed further light on the activation mechanism and the structural and dynamic basis of allosteric regulation in the CRP/FNR family we determined the crystal structures of apo Cg-GlxR at a resolution of 2.5 Å. In addition, the regulator bound to cyclic AMP was solved in two crystal forms diffracting to 2.38 and 1.82 Å resolution. The comparison of apo and cAMP-bound Cg-GlxR structures reveals the conformational changes responsible for activating DNA-binding. Furthermore, one crystal form of the cAMP-bound structure crystallized with two independent dimers in the asymmetric unit, whereas the second form contains six monomers in the asymmetric unit allowing for a detailed analysis of the conformational flexibility. In addition, we investigated cAMP and DNA binding of the protein by Isothermal Titration Calorimetry (ITC) and Fluorescence Anisotropy (FA) measurements. Our results suggest that in contrast to apo Ec-CRP, apo Cg-GlxR adopts a well-defined conformation and undergoes a small but significant hinge motion of the DNA-binding domain upon cAMP binding. Once activated Cg-GlxR adopts a conformation suitable for DNA binding. Subtle structural differences between Ec-CRP and Cg-GlxR explain the observed differences in allosteric behavior. Similarly, seemingly small changes in sequence may lead to different dynamic behavior contributing to allosteric regulation.

## Materials and Methods

### Protein Expression and Purification

Cloning, expression and purification were performed as described previously. Briefly, Cg-GlxR was produced using *E. coli* BL21 (λDE3) transformed with the expression plasmid pET16b-glxr [34], [35]. Pre-cultures in 150 mL LB media supplemented with kanamycin (50 µg/L) were cultivated for 8 h at 37°C and then inoculated 1∶100 in 6 L of autoinducing media for overnight growth at 37°C [36]. Bacteria were harvested by centrifugation at 4,500 g for 15 minutes at 4°C, re-suspended in 50 mL wash buffer (20 mM TrisHCl pH 8, 2 mM EDTA), pelleted at 5,500 g for 30 minutes and stored at −80°C. Pellets were then thawed and re-suspended in lysis buffer (50 mM KH_2_PO_4_ pH 7.8, 200 mM KCl, 20% (v/v) glycerol, 2 mM 1-thioglylcerol, 1 tablet of Roche protease inhibitor cocktail). Cells were lysed by sonication and debris was removed by centrifugation at 50,000 g for 1 h at 4°C. The His_6_-tagged protein was isolated using Ni-NTA-agarose (Invitrogen) where the protein was eluted with 10 mL of lysis buffer containing 150 mM imidazole. The protein was further purified using an Akta Explorer with a Superdex75 size exclusion column using 50 mM KH_2_PO_4_ pH 7.8, 200 mM KCl, 5% glycerol and 2 mM 1-thioglycerol. Peak fractions were pooled and analysed by SDS PAGE and mass spectrometric analysis (data not shown). GlxR in complex with cAMP was purified from IPTG (1 mM) induced *E. coli* JM109 (λDE3) cells containing the pETCRP plasmid [37]. Cells were grown at 37°C in LB medium supplemented with 50 µg/L kanamycin. The protein was purified from cell-lysate using Ni-NTA agarose (Invitrogen) according to published protocols [37]. Further purification was accomplished with a gel filtration column (GE Healthcare). The sample was finally incubated with cAMP in 10 times molar excess prior to crystallisation.

### Isothermal Titration Calorimetry

Apo Cg-GlxR was first dialyzed overnight against 100 mM KH_2_PO_4_ pH 7.8, 200 mM KCl, 2 mM 1-thioglycerol at 4°C. Protein sample and buffer for the cAMP solution were degassed under vacuum. cAMP concentrations were calculated using the Lambert-Beer Law and a molar extinction coefficient of 14,650 M^−1^ cm^−1^ at 259 nm. Data was generated using an iTC200 (MicroCal) by one 0.3 µL injection, followed by 10 sequential 0.5 µL and 24 1 µl injections of 20 mM cAMP into 202 µL 165 µM protein. Data for the first injection was routinely discarded as this is affected by diffusion between the syringe and the protein solution during equilibration prior to data collection. Ligand binding was described with a two-site binding model.

### Fluorescence Anisotropy Measurements

Anisotropy was measured on a Carey Eclipse Fluorescence Spectrophotometer (Agilent Technologies) fitted with polarizing filters (λ_em_ = 520 nm, λ_ex_ = 495 nm, bandwidth = 5 nm, averaging time 20 s). Anisotropy was determined in the presence of 5′-FITC end-labeled oligonucleotide (FITC-5′-ATTAATGTGAGTTAGCTCACTCATTA-3′) and unlabeled oligonucleotide (5′-TAATGAGTGAGCTAACTCACATTAAT-3′). Oligonucleotides were annealed by heating to 90°C for 5 minutes in ultra pure water before cooling slowly to room temperature. Experiments were performed by titrating 2 µL aliquots of 200 µM Cg-GlxR into 5 nM labelled oligonucleotide in 1 mL buffer (100 mM KH_2_PO_4_ pH 7.8, 200 mM KCl, 1 mM 1-thioglycerol), in the presence of 0 or 1.5 mM cAMP. Anisotropy was calculated using WinFLR software (Agilent Technologies).

### Crystallisation and Data Collection

Initial screens of apo cg-GlxR were set up with an Innovadyne Screenmaker using 100 nL and 200 nL protein solution at approximately 10 mg/mL in sitting drops using commercially available crystallisation screens. Follow-up experiments were performed using standard hanging-drop setup. The best crystals were obtained with 100 mM Na/K phosphate, 25% (v/v) propandiol, 10% (v/v) glycerol. Crystals were cryoprotected using a 1∶1 mixture of the reservoir solution and 50% glycerol solution and frozen in liquid [38]. All diffraction data were collected at the Diamond beam line I04 and processed using XDS [39]. Holo Cg-GlxR was crystallised using hanging drop vapor diffusion methods with the following buffer composition: 0.1M Morpheus buffer 3 (Tris/Bicine) at pH 8.5; 30% Morpheus GOL_P4K (glycerol, PEG4000), Morpheus Alcohols Mix at 0.12M (Molecular Dimensions). Diffraction data were collected at the Swiss Light Source beam line X06D and processed with XDS [39].

### Structure solution, refinement and analysis

Crystal form II of cAMP-bound Cg-GlxR was first solved by molecular replacement with PHASER [40] using the structure of CRP from *M. tuberculosis* [41] as a search model. Crystal form I was obtained later and solved with the structure from crystal form II. The apo form of the protein was solved with PHASER using the holo Cg-GlxR structure as a search model. Both structure were completed by iterative cycles of model building using COOT [42] and crystallographic refinement with REFMAC [43], and validated using the various tools in COOT and Procheck [44]. For the apo Cg-GlxR structure 92.9% of all residues are in the preferred region, 5.5% in the allowed regions. Holo Cg-GlxR present 95% in preferred and a further 3.9% in allowed region Those residues that lie in outlier regions are located in flexible and/or poorly defined loop regions. Further crystallographic data are summarized in Table 1. Atomic coordinates and structure factors have been deposited at the Protein Data Bank, Research Collaboratory for Structural Bioinformatics, Rutgers University, New Brunswick, NK with pdb codes 3R6S and 4CYD for holo Cg-GlxR and 4BYY for apo Cg-GlxR.

**Table 1:** Data collection and refinement statistics.

Molecule:	Apo Cg-GlxR	Holo Cg-GlxR - I	Holo Cg-GlxR - II
Beamline	DLS I04	DLS I04	SLS X06DA
Wavelength [Å]	0.9795	0.9163	1.0
Space group	C2	P2_1_	P3_1_21
a [Å]	95.64	63.93	111.25
b [Å]	59.95	102.66	111.25
c [Å]	90.88	82.22	186.95
α,β, χ [°]	90, 110.0, 90	90, 108.5, 90	90, 90, 120
resolution range [Å]	85-2.48	29.41 - 1.82	67-2.38
Wilson B [Å^2^]	50.0	37.9	42.6
R_merge_ [%]*	0.042 (0.724)	0.045 (0.52)	0.088 (0.705)
Completeness [%]	99.6	98.4	99.4
R [%]	0.207	0.192	0.210
R_free_ [%]**	0.285	0.246	0.288
No. of residues chain A	1–226	3–15,17 169,171–227	3–227
No. of residues chain B	3–164, 178–217	3–227	3–227
No. of residues chain C		3–227	3–226
No. of residues chain D		3–227	3–226
No. of residues chain E			3–227
No. of residues chain F			3–64, 68–227
Average B-factors per chains [Å^2^]***	A: 47.6; B: 52.1 H_2_O: 76.8		A: 41.1; B: 46.3;C: 43.9; D: 47.8; E; 35.7 F: 41.7; H_2_O:39.2; cAMP: 32.4
No. of cAMP	0	2	6
No. of water	72	2	161
rmsd bond length [Å]	0.012	0.019	0.016
rmsd bond angles[°]	1.67	1.90	1.72
PDB code	4BYY	4CYD	3R6S

*number in brackets reveal to the last resolution shell.

**Rfree is based over a subset of 5% of reflection that have not been used through the refinement [59].

***Average B-factors without TLS contributions.

## Results and Discussion

### Overall structure of Cg-GlxR

**Figure 1:**
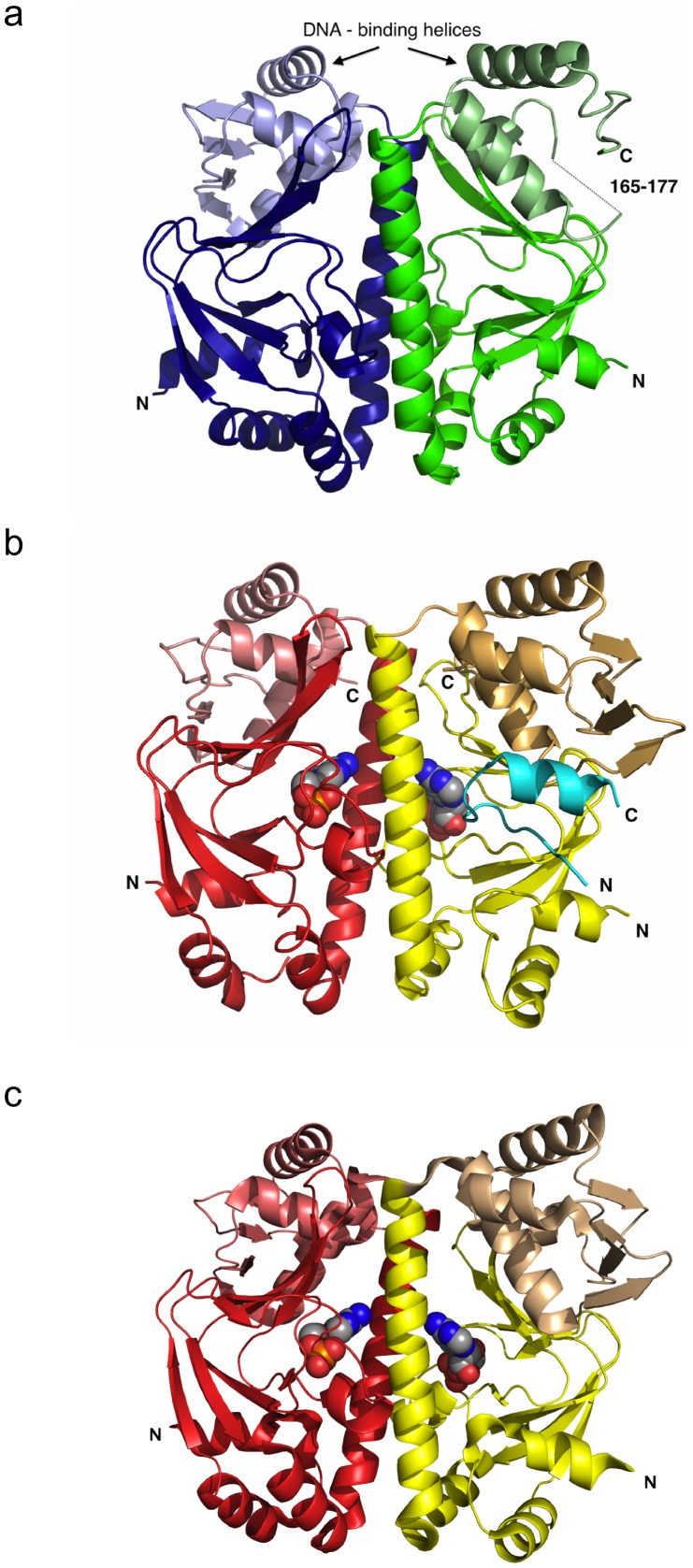
Crystal structures of Cg-GlxR. a) Ribbon representation of the apo Cg-GlxR homodimer. View shown approximately perpendicular to the non-crystallographic 2-fold axis with the DNA binding domains at the top. The two DNA-recognition helices are approximately parallel to each other. The dimerisation and cAMP binding domain is located at the bottom of the figure. The two protein chains are depicted in blue and green, respectively, with the DNA-binding domains in lighter shade b) Ribbon representation of one holo Cg-GlxR dimer in crystal form I shown in the same orientation with each protein chain in red and yellow with the DNA-binding domains on lighter shades of the same color. The N-terminal tag is visible in one monomer of each dimer and depicted in cyan. c) Ribbon diagram of one representative dimer of holo Cg-GlxR in crystal form II using the same color code. The ligand, cyclic AMP is shown in a ball-and-stick representation.

**Figure 2:**
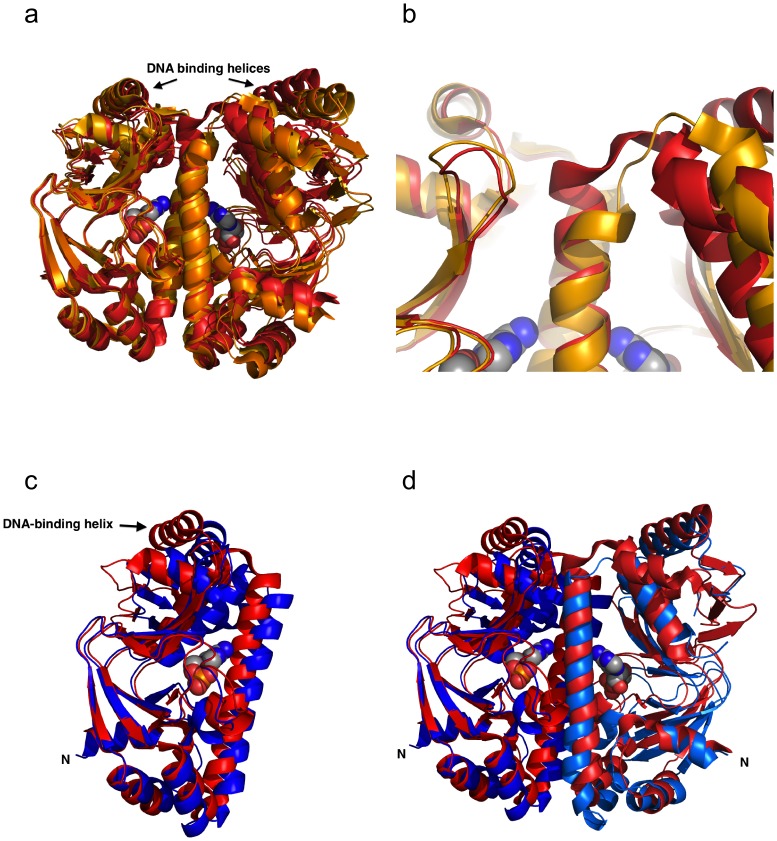
Comparison of Cg-GlxR crystal structures. a) Least-squares superposition of the four crystallographically independent holo Cg-GlxR dimers in crystal form II. Two distinct conformations shown in red and orange, respectively can be clearly distinguished. b) Close-up view of the holo Cg-GlxR dimers displaying the differences at the top of the dimerisation helix. The two dimers in crystal form I adopt a conformation similar to the one shown in red. c) Superposition of apo Cg-GlxR monomer (in blue) with holo Cg-GlxR (in red). View shown approximately perpendicular to the non-crystallographic 2-fold dimer axis. d) Superposition of the apo Cg-GlxR (in blue) dimer with holo Cg-GlxR (in red). In all least-squares superpositions only the ligand binding domain on the left-hand side of the picture was used to calculate the transformation matrix with COOT [42] that was then applied to the entire protein dimer.

The crystal structures of apo and holo Cg-GlxR were determined independently at resolutions ranging from 2.5 to 1.82 Å (Table 1). Apo Cg-GlxR crystallises in the monoclinic space group C2 with one homo-dimer in the asymmetric unit (Figure 1a). Chain A shown on the left-hand side in blue is well defined with residues 1–226 (out of 227) present in the model, chain B (green) shows a larger degree of flexibility, and hence the model contains residues 3–164 and 178–217. The loop region of residues 165–177 that forms part of an anti-parallel β-sheet shows no density and was consequently not included. In addition, the model contains two phosphate ions with an occupancy of 0.5 in the cAMP binding site, two glycerol molecules at the protein surface and 72 water molecules. Crystal form I of cAMP bound Cg-GlxR crystallizes with two independent dimers in the asymmetric unit. Each monomer contains one cAMP in the binding pocket (Figure 1b). In addition, each dimer contains a portion of the tag used for purification (20 residues, shown in cyan in Figure 1b) providing crystal contacts. Crystal form II of holo Cg-GlxR in contrast, crystallizes with six independent protein chains, two complete dimers and two monomers on crystallographic 2-fold axes in the asymmetric unit. Each homodimer contains two cAMP molecules bound in the ligand-binding pocket of the monomer (Figure 1c). The overall structures of apo and holo Cg-GlxR exhibit the familiar domain structure seen in CRP from *E. coli* and *M. tuberculosis* [21], [41], [45]. Each monomer is composed of an N-terminal ligand-binding domain, followed by the central long helix, which provides the dimer interface and contributes to cAMP binding, and the C-terminal DNA-binding domain with its characteristic DNA-binding helix-turn-helix motif. The two independent protein chains in the apo Cg-GlxR structure adopt a similar conformation with an rmsd of 1.36 Å for 200 equivalent Cα-atoms (all least-squares superpositions were calculated using the RAPIDO server [46], Table 2). It is noteworthy that the DNA binding domains show a higher degree of flexibility as indicated by higher B-factors and a partial disorder of two external β-strands (Figure 1a). In contrast, all DNA binding domains are well defined in all ten independent molecules of the two holo Cg-GlxR crystal forms, adopting a similar conformation with rmsd's for all equivalent Cα-atoms ranging from 0.5 to 1.5 Å. The main differences between the crystallographically independent molecules occur through a rearrangement of the protein monomers in each dimer with respect to each other. This mobility becomes apparent when only the ligand-binding domain of one chain (residues 3–119) is used to calculate the superposition matrix of all holo GlxR dimers (Figure 2a, 2b). The monomers that are used to calculate the superposition (left-hand side) fit very well with rmsds of less than 1 Å, however, two distinct dimer arrangements shown in red and pale red can clearly be seen. The conformational differences are due to the dissimilar environments in the crystals representative of the conformational flexibility of the active regulator in solution.

**Table 2:** Comparison of holo and cAMP bound Cg-GlxR in crystal form I and II.

	apo A	apo B	holo I A	holo I B	holo I C	holo I D	holo II A	holo II B	holo II C	holo II D	holo II E	holo II F
apo A*		1.36 *200*	1.96 *222*	1.66 *224*	2.13 *224*	2.02 214	2.05 *224*	1.86 *224*	1.75 *224*	2.17 *224*	1.98 *221*	2.06 *220*
apo B	1.36 *(1)* **		2.40 *199*	2.14 *201*	2.66 *201*	2.42 *196*	2.39 *200*	2.13 *200*	2.09 *187*	2.50 *200*	2.40 *199*	2.37 *196*
holo I A	1.02 *(2)*	0.76 *(3)*		0.69 *223*	1.01 *222*	0.65 *214*	0.65 *222*	0.77 *223*	0.84 *222*	0.71 *222*	1.25 *222*	1.17 *218*
holo I B	1.02 *(3)*	1.25 *(3)*	0.66 *(1)*		0.88 *225*	0.94 *212*	0.76 *225*	0.69 *225*	0.46 *224*	0.91 *224*	1.19 *223*	1.25 *220*
holo I C	1.09 *(3)*	0.86 *(3)*	0.82 *(1)*	0.71 *(1)*		1.27 *211*	1.16 *225*	1.26 *225*	0.98 *224*	1.24 *224*	1.46 *224*	1.60 *221*
holo I D	1.24 *(2)*	0.98 *(2)*	0.62 *(1)*	0.83 *(1)*	0.95 *(1)*		0.73 *213*	0.84 *214*	1.03 *214*	0.78 *212*	1.44 *211*	1.19 *206*
holo II A	1.08 *(3)*	0.90 *(3)*	0.63 *(1)*	0.71 *(2)*	0.75 *(1)*	0.63 *(1)*		0.63 *225*	0.86 *224*	0.57 *224*	1.42 *224*	1.10 *219*
holo II B	1.23 *(2)*	0.82 *(3)*	0.74 *(1)*	0.69 *(1)*	0.83 *(2)*	0.78 *(1)*	0.63 *(1)*		0.71 *223*	0.75 *224*	1.25 *225*	1.10 *221*
holo II C	1.06 *(3)*	0.95 *(3)*	0.74 *(1)*	0.46 *(1)*	0.72 *(1)*	0.89 *(1)*	0.86 *(1)*	0.71 *(1)*		1.00 *223*	1.06 *222*	1.27 *221*
holo II D	1.17 *(3)*	0.83 *(3)*	0.61 *(1)*	0.80 *(1)*	0.87 *(1)*	0.70 *(1)*	0.57 *(1)*	0.75 *(1)*	1.0 *(1)*		1.46 *222*	1.13 *218*
holo II E	1.20 *(3)*	0.86 *(3)*	0.80 *(1)*	0.85 *(1)*	0.83 *(2)*	0.96 *(1)*	1.42 *(1)*	1.25 *(1)*	1.06 *(1)*	1.46 *(1)*		0.85 *222*
holo II F	1.48 *(3)*	0.81 *(3)*	0.86 *(1)*	0.81 *(1)*	0.84 *(2)*	0.84 *(1)*	1.23 *(1)*	1.10 *(1)*	1.27 *(1)*	1.13 *(1)*	0.85 *(1)*	

Root-mean-squares deviations after least-squares superpositioning of individual protein chains.

*In the right upper half of the table, the upper number denotes the rmsd in Å assuming rigid molecules. The number of equivalent Cα-atoms used for the least-squares superposition is given in italics.

**The left lower half of the table gives the rmsd in Å for a flexible superposition where the number of domains is given in brackets. These rmsds are generally slightly lower even if only one rigid domain has been used compared to the full alignment because the single rigid domain identified is generally slightly smaller.

### Comparison of apo and holo Cg-GlxR

Comparing the apo and holo GlxR structures shows that the rmsds for all equivalent Cα-atoms in each chain treated as rigid-body are significantly higher ranging from 1.8 to 2.2 Å for chain A and 2.0 to 2.5 Å for chain B, respectively (Table 2). In order to identify the conformational changes induced, the ligand-binding domain (residues 3–119) of one monomer was again used to calculate the least-squares superposition transformation matrix, which was then applied to the protein dimers. Whereas the ligand binding domains of apo and holo GlxR superimpose very well with no significant changes within the domain as indicated of rmsds ranging from 0.8–1.5 Å for flexible superpositions (Table 2), the position of the DNA-binding domain with respect to the ligand binding domain in the same monomer changes significantly. This motion becomes particularly apparent when ligand-binding domain of one monomer is used to calculate the superposition matrix (Figure 2c and 2d). The two DNA-recognition helices in apo Cg-GlxR (blue) and holo Cg-GlxR (red) have moved significantly with respect to each other. Equivalent Cα-positions at the tip of this helix move by up to 10 Å upon ligand binding. These differences maybe sufficient to explain the difference of apo and holo Cg-GlxR in DNA-binding capability.

### Ligand and DNA binding

**Figure 3:**
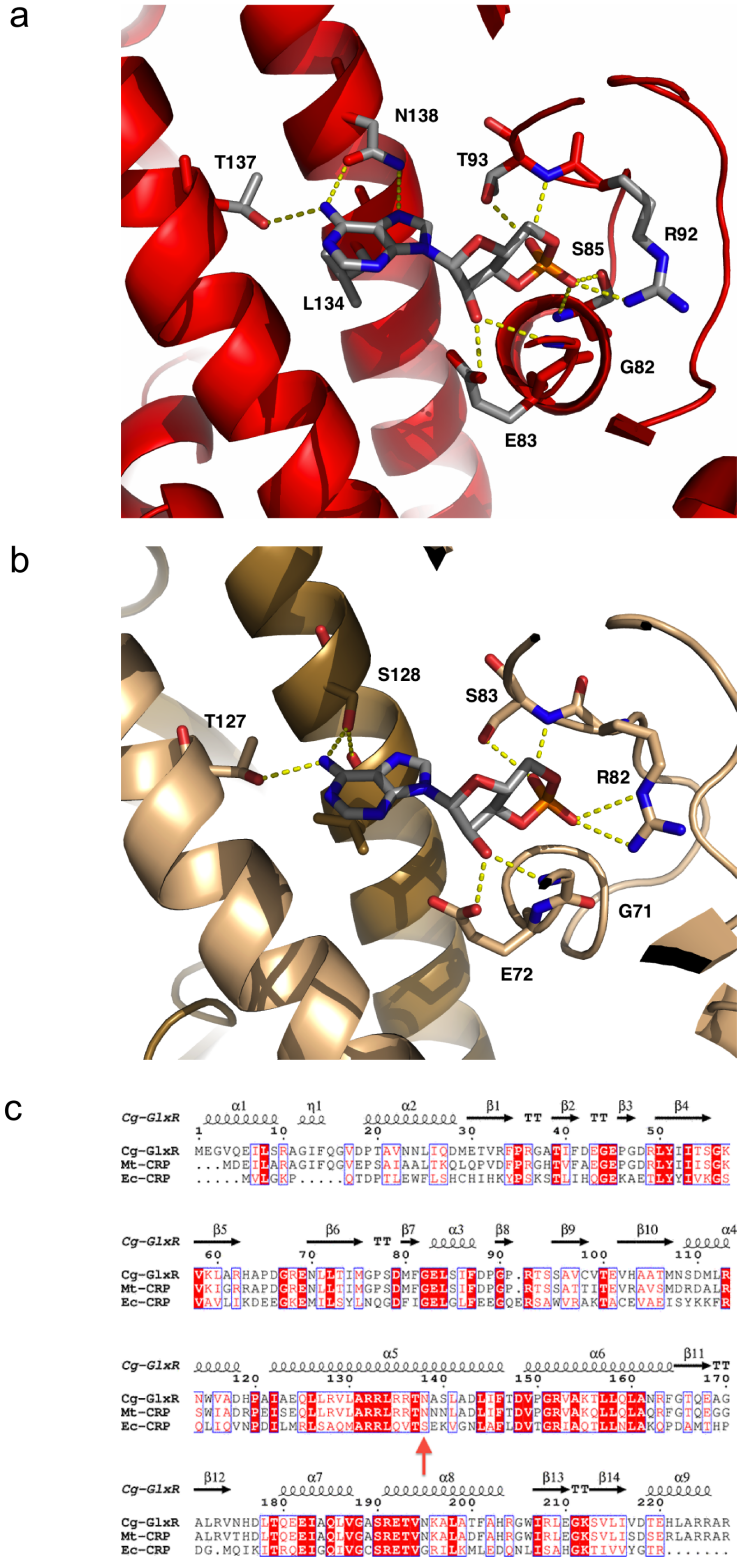
Close-up of the co-factor binding site. a) Close-up of the cAMP binding site in Cg-GlxR depicted in red with the cAMP shown in a ball-and-stick representation; It should be noted that the binding sites in both monomers are identical (b) Close-up of the cAMP binding site in Ec-CRP. c) Sequence alignment of Cg-GlxR compared to Ec-CRP and Mt-CRP generated with ClustalW [57] and ESPRIPT [58] with the secondary structure assignment based on the crystal structure of Cg-GlxR presented here. Residue N138 is highlighted with a red arrow.

**Figure 4:**
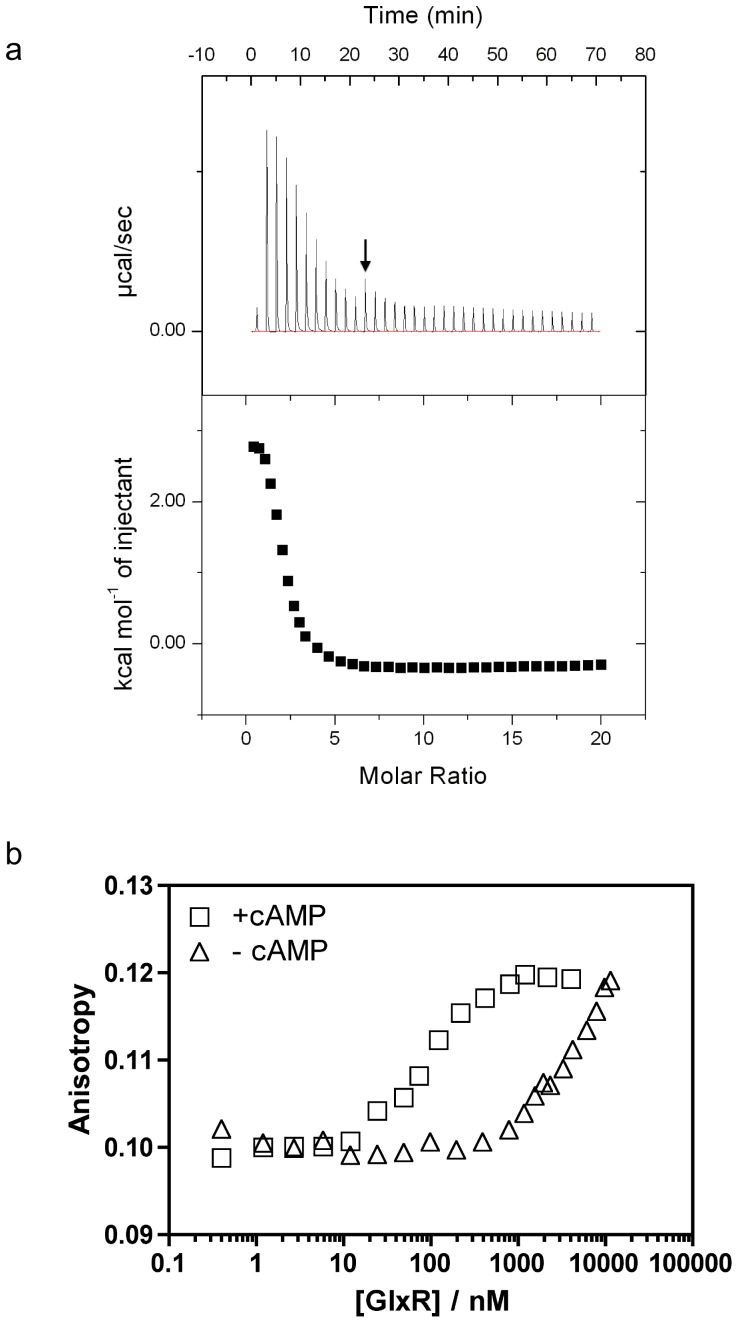
Biophysical Characterisation of co-factor and DNA binding of Cg-GlxR. a) Isothermal titration calorimetry traces (upper panel) and binding isotherms (lower panel) for the titration of cAMP to Cg-GlxR. The arrow indicates the switch from 0.5 µl to 1 µl injections. K_D1_ = 1.7·10^−5^ mol/l; K_D2_ = 1.3·10^−4^ mol/L. b) DNA-binding: Fluorescence Anisotropy data showing the cAMP dependent DNA recognition.

The Cg-GlxR homo-dimer contains two binding sites for its activator cAMP (Figure 3a). As reported previously, Cg-GlxR shows pronounced negative allosteric behavior where binding of the first ligand reduces the affinity of the second - structurally identical - site in the homo-dimer (K_D1_ = 1.7·10^−5^ mol/l; K_D2_ = 1.3·10^−4^ mol/L) [32]. Allostery in Ec-CRP arises from the fact that the first binding event is enthalpically and entropically favored (ΔH_1_ = −2.0 kcal/mol, −TΔS_1_ = −4.9 kcal/mol) presumably due to the large motion of the DNA-binding domains whereas the second binding event is enthalpically disfavored (ΔH_1_ = +8.2 kcal/mol) but entropically favored (−TΔS_1_ = −14.8 kcal/mol). The two binding events are thus highly correlated leading to large changes in dynamic behavior supporting the notion of a largely dynamically driven process of allostery [47]. The structural details presented here support the thermodynamic data where the two binding events of cAMP to Cg-GlxR show similar enthalpic and entropic contributions [32]. Both binding events show a positive enthalpic contribution but are entropically favored. Negative allostery arises from the fact that ΔH_1_ is smaller (2.9 kcal/mol) than ΔH_2_, (4.0 kcal/mol) and −TΔS_1_ (−10.1 kcal/mol) slightly more negative than −TΔS_2_ (−9.5 kcal/mol). The small differences in enthalpic and entropic contributions to the free enthalpies are consistent with the relatively small structural changes observed in the Cg-GlxR structures presented here. The dynamic contributions to allostery may arise from a tightening upon binding of the first cAMP molecule leading to a smaller entropic gain for the second binding event. The crucial difference in the cAMP-binding site between Cg-GlxR and Ec-CRP is the fact the Ser-128 (numbering according to the *E. coli* CRP sequence) is substituted by Asn-138 (Figure 3b). Both residues bind to cAMP in the other binding site (chain A to cAMP bound in monomer B and vice versa) however, only serine is able to form an additional H-bond back to the backbone carbonyl oxygen of Leu124 thus providing an additional route of linking the two separate binding events of cAMP (Figure 3c). This notion is supported by results from Mt-CRP, which shares the asparagine at this position and previous work on Ec-CRP where the Ser128 to Ala mutation abolished allosteric behavior [48], [49]. In addition, the side chain of asparagine can form two H-bonds to N6 and N7 of cAMP, respectively, thus providing a gain of enthalpic contribution as indicated by a significantly smaller value of ΔH_2_ in Cg-GlxR compared to Ec-CRP where the first binding event is not accompanied by a large conformational change. ITC experiments using a higher cAMP concentration were performed to investigate a possible low-affinity third binding site as reported for Ec-CRP. Unlike Ec-CRP [50], Cg-GlxR does not appear to possess a low-affinity second binding site per protein monomer (Figure 4a). Once activated, cAMP-bound GlxR's central role is to bind its target DNA sequences. DNA-binding was therefore further investigated by Fluorescence Anisotropy measurement (Figure 4b). These experiments showed an increase of affinity to DNA by more than 100-fold from a dissociation constant of 8.3 µM to 87 nM upon cAMP binding. These values compare well with binding affinities reported for Ec-CRP [51].

### Comparison of Cg-GlxR with Mt-CRP

**Figure 5:**
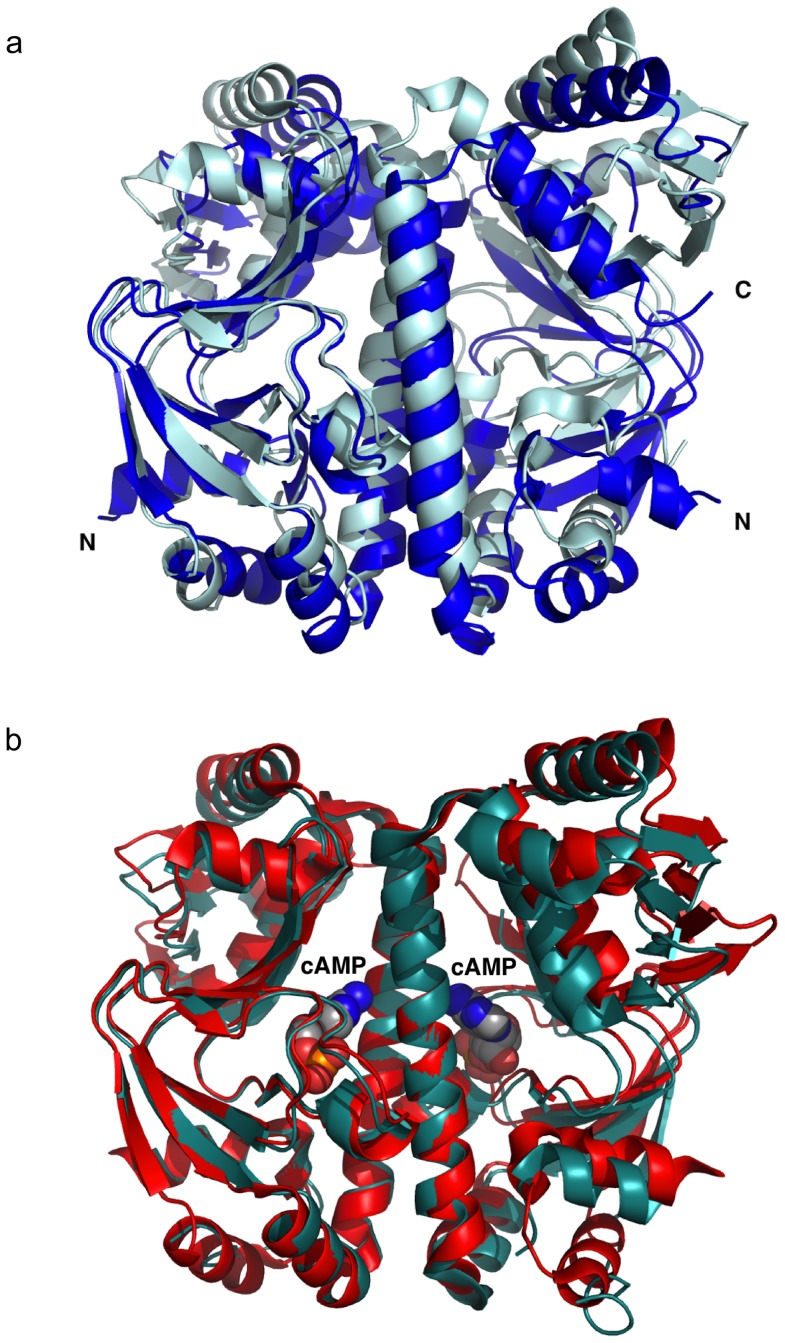
Comparison of Cg-GlxR with Mt-CRP. a) Least-squares superposition of apo Cg-GlxR shown in blue with apo Mt-CRP (PDB code: 3D0S) b) Superposition of holo Cg-GlxR depicted in red with apo Mt-CRP (PDB code: 3I54) in cyan.

Although the apo- and holo-structures of a number of members of the CRP/FNR family have been structurally characterized, our understanding of the structural and dynamics changes caused by ligand-binding is still limited [52]. We therefore compared the structures of Cg-GlxR presented here with the structures of the closest orthologue, Mt-CRP (sequence identity of 79%) as well as with Ec-CRP. The cAMP-free CRP dimer from *M. tuberculosis* has been found to exhibit significant asymmetry between the monomers which has been suggested to be responsible for inactivation [27], however, this profound asymmetry was not observed in an independently determined different crystal form and may therefor be an artifact of crystallisation [53]. The apo Cg-GlxR structure presented here shows some degree of asymmetry as indicated by missing loop regions in one of the two monomers, however, the two monomers superimpose very well with an rmsd of 1.36 Å for all equivalent Cα-atoms. Consequently, the superposition of apo Cg-GlxR with apo Mt-CRP results in relatively large rmsd's ranging from 2.0–3.3 Å (Table 3). As expected these larger differences are due to subunit and domain motions of the C-terminal DNA-binding with respect to the N-terminal ligand-binding domain as illustrated in Figure 5a where only one ligand-binding domain was used to calculate the superposition for the entire dimer. Remarkably, the holo structures of Cg-GlxR and Mt-CRP superimpose with significantly lower rmsds ranging from 0.9 to 1.5 Å (Table 4) as illustrated in Figure 5b. Clearly, the activated cAMP-bound forms of Cg-GlxR and Mt-CRP adopt a more defined conformation better suited for DNA recognition. Apo Cg-GlxR and Mt-CRP in contrast show larger conformational flexibility as illustrated by a larger degree of disorder in the DNA-binding domains and the difference in structures depending on crystallization conditions and crystal packing environments. These finding underline the notion that changes in dynamics play an important role in the activation of DNA binding activity.

**Table 3:** Comparison of apo Cg-GlxR with apo Mt-CRP.

	Apo Mt-CRP (A)	Apo Mt-CRP (B)
Apo Cg-GlxR (A)	2.27* (221)	3.27 (215)
Apo Cg-GlxR (B)	2.71 (197)	3.31(201)

Root-mean-squares deviations after least-squares superpositioning of protein chains.

*The first number denotes the rmsd in Å for a rigid superpositioning of protein chains. The number of brackets gives the number of Cα-atoms used.

**Table 4:** Comparison of holo nCg-GlxR with cAMP-bound forms of Mt-CRP (PDB code 3I54), Ec-CRP (PDB code: 1G6N) and Ec-CRP bound to DNA (PDBP code: 1J59).

	holo Mt-CRP (A)*	holo Mt-CRP (B)	holo Ec-CRP (A)	holo Ec-CRP (B)	DNA holo Ec-CRP A	DNA holo Ec-CRP B
holo Cg-GlxR (A)	0.92 (216)	0.89 (206)	2.02 (183)	3.79 (181)	1.81 (183)	1.97 (180)
holo Cg-GlxR (B)	0.95 (219)	1.12 (206)	1.99 (194)	3.80 (186)	1.85 (191)	1.91 (188)
holo Cg-GlxR (C)	1.11 (218)	1.34 (210)	2.31 (190)	4.10 (188)	2.20 (194)	2.34 (193)
holo Cg-GlxR (D)	1.15 (207)	1.05 (201)	2.14 (181)	3.77 (183)	1.97 (182)	2.14 (187)
holo Cg-GlxR (A)	1.08 (218)	1.09 (207)	2.02 (192)	3.69 (186)	1.84 (187)	1.95 (188)
holo Cg-GlxR (B)	0.97 (218)	1.07 (207)	2.18 (194)	3.87 (182)	1.98 (189)	2.11 (189)
holo Cg-GlxR (C)	0.99 (218)	1.21 (207)	2.28 (194)	3.95 (188)	2.01 (190)	2.10 (189)
holo Cg-GlxR (D)	1.10 (218)	0.97 (207)	2.01 (189)	3.69 (183)	1.87 (189)	2.02 (188)
holo Cg-GlxR (E)	1.16 (217)	1.45 (206)	2.48 (193)	4.22 (183)	2.32 (190)	2.40 (188)
Holo Cg-GlxR (F)	1.31 (213)	1.54 (208)	2.32 (189)	3.96 (182)	2.15 (187)	2.27 (186)

The four independent monomers of crystal form I are in the first four rows and, the six independent monomers of crystal form II in the remaining six rows.

*The first number denotes the rmsd in Å for a rigid superpositioning of protein chains using RAPIDO. The number of brackets gives the number of Cα-atoms used.

### Comparison of Cg-GlxR with Ec-CRP

**Figure 6:**
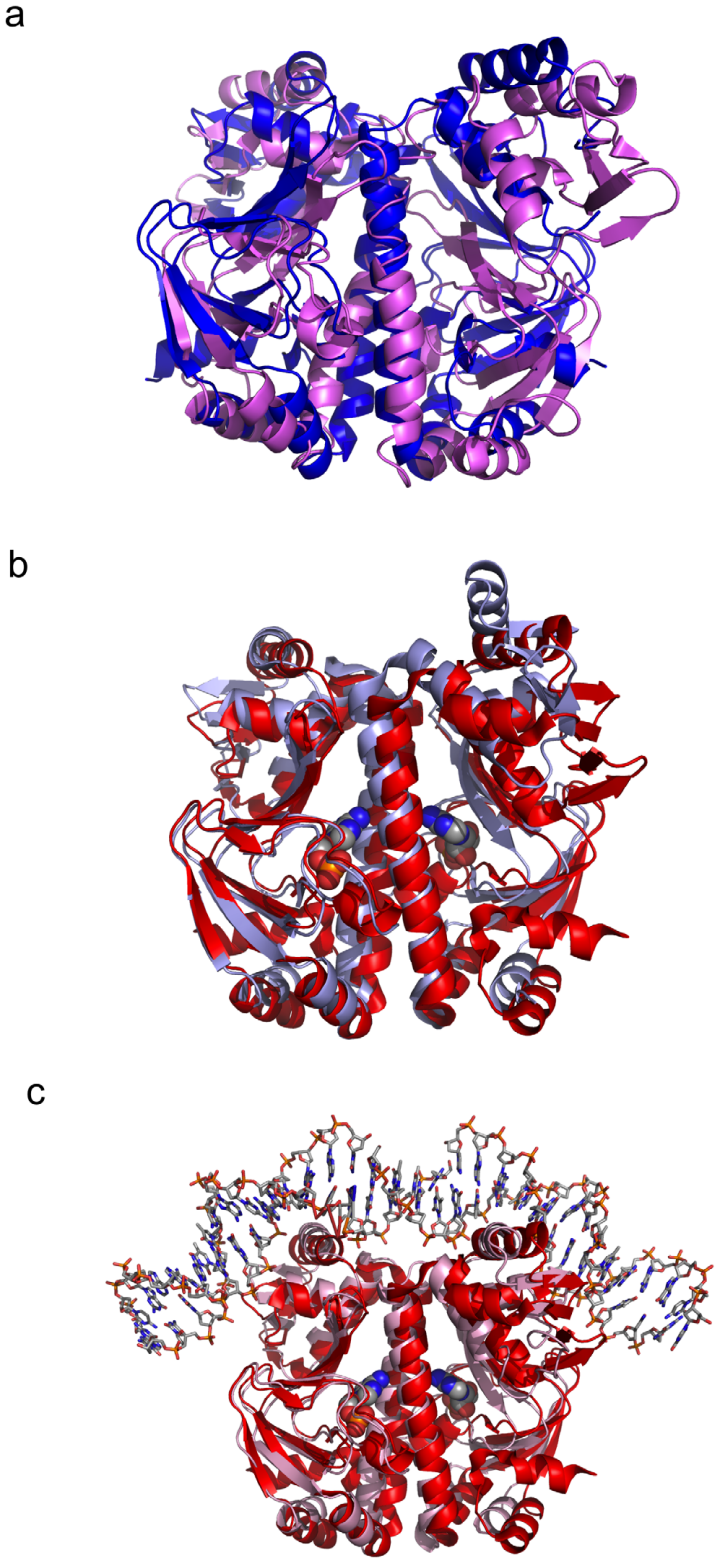
Superpositions of the crystal structure of Cg-GlxR with Ec-CRP. a) apo Cg-GlxR in blue with the NMR structure of Ec-CRP in magenta (PDB code 2WC2) b) holo Cg-GlxR (in red) superimposed on cAMP bound Ec-CRP (PDB code: 1G6N) shown in light blue; c) holo Cg-GlxR (in red) superimposed on cAMP-bound Ec-CRP as bound to DNA (PDB code: 1J59) shown in pink.

In order to assess if the findings presented here based on the structures can be generalized for the diverse CRP/FNR family, we compared the structures of holo-GlxR with Ec-CRP in its cAMP-bound state [50] and in complex with DNA [23]. It should be noted that the apo-form of Ec-CRP has only been solved at medium resolution and shows a significant degree of disorder [26] and was therefore not included in this comparison. Instead we used on representative structure from the NMR ensemble of apo Ec-CRP (pdb code 2WC2 [25]) for least-squares superpositioning (Figure 6a). As expected the overall structures are similar but the NMR structures shows significant motion of the individual domains with respect to each other which could be due to the fact that the NMR structures are not constrained by the crystal lattice. The same argument could account for the observation that the C-terminal loop of the long dimerization helix appears to be more flexible in apo Ec-CRP compared to apo Cg-GlxR despite a high level of sequence conservation in this area. A more detailed comparison can be performed using the multiple crystal structures of holo Cg-GlxR with holo Ec-CRP. As expected given the low sequence identity the overall structures are similar but differ far more on a detailed level. The rmsds for each of the six Cg-GlxR chains with Ec-CRP range from 2.0 to 4.2 Å indicating large conformational changes (see Table 4). As the two separate domains superimpose very well with rmsd of 1.0–1.2 Å when motions of 2 (or in some cases 3) domains are taken into account, we used the same least-squares superposition of one N-terminal ligand binding domain to superimpose equivalent dimers of Cg-GlxR and Ec-CRP. The results shown in Figure 6b indicate that there is a small re-orientation of the monomer-to-monomer position combined with a larger re-arrangement of the DNA-binding domain with respect to the ligand-binding domain. Remarkably, this re-arrangement is not symmetric with a much larger difference seen in the second monomer (depicted on the right-hand side of protein dimer in Figure 6b). However, it is important to note that the differences in orientation of domains in two identical protein chains may be due to crystal packing effects. This notion is supported by the fact that the cAMP-bound Cg-GlxR structure superimposes significantly better with the activated Ec-CRP conformation found in its active DNA-bound form (Figure 6c). The rmsds in this case range only from 1.8–2.4 Å. It is therefore highly likely that the ligand-bound form of Cg-GlxR structure represents the *active* state capable of DNA-recognition.

## Conclusions

The structures of apo and cAMP-bound GlxR from *C. glutamicum* presented here provide a detailed picture of the structural changes required for DNA-binding. As seen in a number of homo-dimeric bacterial regulator proteins, the re-arrangement of the DNA-binding domains with respect to the ligand-binding and dimerisation domain(s) is at least partially responsible for activation [54]–[56]. In this case cAMP-binding re-orientates the position of the DNA-binding domains in the Cg-GlxR dimer to optimize DNA recognition. The fact that the cAMP-bound form of Cg-GlxR crystallises in two crystal forms with six different dimers allows us to assess the still considerable conformational flexibility of holo-GlxR. The crystal structure provides an ensemble of energetically almost identical conformations where the positions of the DNA-binding domains differ by up to 3 Å for equivalent Cα-positions. Binding to its target DNA sequence will presumably lock the conformation in the most favorable position for forming the ternary cAMP-Cg-GlxR-DNA complex. The structural changes shown here for Cg-GlxR upon cAMP binding, however, are quite distinct from those observed previously for Ec-CRP. Based on the structure determination by NMR a rotation of about 60° of the DNA-binding domain coupled with a coil-to-helix transition has been reported for Ec-CRP [25]. Likewise, the asymmetric homo-dimer observed in one crystal structure of apo Mt-CRP cannot be reproduced here. All Cg-GlxR structures form symmetric homo-dimers. While apo Cg-GlxR is clearly more flexible compared to cAMP-bound form as indicated by partially disordered loops in one of the two independent DNA-binding domains the structural change caused by cAMP binding depicted in Figure 2 is smaller than observed for other members of the CRP/FNR family. Nevertheless, there is a significant movement changing the position of the DNA-binding helix in monomer A with respect to monomer B by up to 10 Å (measured by comparing equivalent Cα-positions of the DNA-binding helix in monomer B). This motion is more than enough to render the apo Cg-GlxR less suited for DNA-binding. It is important to note that, whereas the cAMP-bound form of Ec-CRP does not adopt the optimal conformation for DNA binding, the activated Cg-GlxR structure presented here superimposed better with the DNA-bound form of holo Ec-CRP than with the DNA-free form of holo Ec-CRP (Figure 6c). The biophysical and structural studies determined in this work underline the notion that the mechanisms of allosteric binding and activation of DNA binding differ considerably in the CRP/FNR family without dramatic structural changes. The same 3-dimensional fold is finely tuned using seemingly small structural changes couple with changes in dynamic behavior to achieve the optimal combination of allostery and DNA recognition.

## References

[1] Leuchtenberger W, Huthmacher K, Drauz K (2005) Biotechnological production of amino acids and derivatives: current status and prospects. Appl Microbiol Biotechnol 69:1–8. 16195792 10.1007/s00253-005-0155-y

[2] Utagawa T (2004) Production of arginine by fermentation. J Nutr 134:2854S–2857S discussion 2895S. 15465800 10.1093/jn/134.10.2854S

[3] Vogt M, Haas S, Klaffl S, Polen T, Eggeling L, et al (2014) Pushing product formation to its limit: Metabolic engineering of Corynebacterium glutamicum for L-leucine overproduction. Metabolic Engineering 22:40–52. 24333966 10.1016/j.ymben.2013.12.001

[4] Okino S, Noburyu R, Suda M, Jojima T, Inui M, et al (2008) An efficient succinic acid production process in a metabolically engineered Corynebacterium glutamicum strain. Applied Microbiology and Biotechnology 81:459–464. 18777022 10.1007/s00253-008-1668-y

[5] Wendisch VF, Bott M, Eikmanns BJ (2006) Metabolic engineering of Escherichia coli and Corynebacterium glutamicum for biotechnological production of organic acids and amino acids. Curr Opin Microbiol 9:268–274. 16617034 10.1016/j.mib.2006.03.001

[6] Litsanov B, Brocker M, Bott M (2012) Toward Homosuccinate Fermentation: Metabolic Engineering of Corynebacterium glutamicum for Anaerobic Production of Succinate from Glucose and Formate. Applied and Environmental Microbiology 78:3325–3337. 22389371 10.1128/AEM.07790-11 PMC3346441

[7] Yukawa H, Inui M, Suzuki N (2004) Production of green products from biomass by novel bioprocess. Abstracts of Papers of the American Chemical Society 227:U301–U301.

[8] Vertes AA, Inui M, Yukawa H (2008) Technological options for biological fuel ethanol. Journal of Molecular Microbiology and Biotechnology 15:16–30. 18349547 10.1159/000111989

[9] Blombach B, Riester T, Wieschalka S, Ziert C, Youn JW, et al (2011) Corynebacterium glutamicum Tailored for Efficient Isobutanol Production. Applied and Environmental Microbiology 77:3300–3310. 21441331 10.1128/AEM.02972-10 PMC3126470

[10] Bott M (2007) Offering surprises: TCA cycle regulation in Corynebacterium glutamicum. Trends Microbiol 15:417–425. 17764950 10.1016/j.tim.2007.08.004

[11] Krug A, Wendisch VF, Bott M (2005) Identification of AcnR, a TetR-type repressor of the aconitase gene acn in Corynebacterium glutamicum. J Biol Chem 280:585–595. 15494411 10.1074/jbc.M408271200

[12] Cramer A, Gerstmeir R, Schaffer S, Bott M, Eikmanns BJ (2006) Identification of RamA, a novel LuxR-type transcriptional regulator of genes involved in acetate metabolism of Corynebacterium glutamicum. J Bacteriol 188:2554–2567. 16547043 10.1128/JB.188.7.2554-2567.2006 PMC1428430

[13] Wennerhold J, Krug A, Bott M (2005) The AraC-type regulator RipA represses aconitase and other iron proteins from Corynebacterium under iron limitation and is itself repressed by DtxR. J Biol Chem 280:40500–40508. 16179344 10.1074/jbc.M508693200

[14] Wennerhold J, Bott M (2006) The DtxR regulon of Corynebacterium glutamicum. J Bacteriol 188:2907–2918. 16585752 10.1128/JB.188.8.2907-2918.2006 PMC1446976

[15] Kim HJ, Kim TH, Kim Y, Lee HS (2004) Identification and characterization of glxR, a gene involved in regulation of glyoxylate bypass in Corynebacterium glutamicum. J Bacteriol 186:3453–3460. 15150232 10.1128/JB.186.11.3453-3460.2004 PMC415749

[16] Kohl TA, Baumbach J, Jungwirth B, Puhler A, Tauch A (2008) The GlxR regulon of the amino acid producer Corynebacterium glutamicum: in silico and in vitro detection of DNA binding sites of a global transcription regulator. J Biotechnol 135:340–350. 18573287 10.1016/j.jbiotec.2008.05.011

[17] Toyoda K, Teramoto H, Inui M, Yukawa H (2011) Genome-wide identification of in vivo binding sites of GlxR, a cyclic AMP receptor protein-type regulator in Corynebacterium glutamicum. J Bacteriol 193:4123–4133. 21665967 10.1128/JB.00384-11 PMC3147711

[18] Jungwirth B, Sala C, Kohl TA, Uplekar S, Baumbach J, et al (2013) High-resolution detection of DNA binding sites of the global transcriptional regulator GlxR in Corynebacterium glutamicum. Microbiology 159:12–22. 23103979 10.1099/mic.0.062059-0

[19] Han SO, Inui M, Yukawa H (2007) Expression of Corynebacterium glutamicum glycolytic genes varies with carbon source and growth phase. Microbiology 153:2190–2202. 17600063 10.1099/mic.0.2006/004366-0

[20] Deutscher J (2008) The mechanisms of carbon catabolite repression in bacteria. Curr Opin Microbiol 11:87–93. 18359269 10.1016/j.mib.2008.02.007

[21] McKay DB, Steitz TA (1981) Structure of catabolite gene activator protein at 2.9 A resolution suggests binding to left-handed B-DNA. Nature 290:744–749. 6261152 10.1038/290744a0

[22] Schultz SC, Shields GC, Steitz TA (1991) Crystal structure of a CAP-DNA complex: the DNA is bent by 90 degrees. Science 253:1001–1007. 1653449 10.1126/science.1653449

[23] Parkinson G, Wilson C, Gunasekera A, Ebright YW, Ebright RH, et al (1996) Structure of the CAP-DNA complex at 2.5 angstroms resolution: a complete picture of the protein-DNA interface. J Mol Biol 260:395–408. 8757802 10.1006/jmbi.1996.0409

[24] Passner JM, Steitz TA (1997) The structure of a CAP-DNA complex having two cAMP molecules bound to each monomer. Proc Natl Acad Sci U S A 94:2843–2847. 9096308 10.1073/pnas.94.7.2843 PMC20284

[25] Popovych N, Tzeng SR, Tonelli M, Ebright RH, Kalodimos CG (2009) Structural basis for cAMP-mediated allosteric control of the catabolite activator protein. Proc Natl Acad Sci U S A 106:6927–6932. 19359484 10.1073/pnas.0900595106 PMC2678429

[26] Sharma H, Yu S, Kong J, Wang J, Steitz TA (2009) Structure of apo-CAP reveals that large conformational changes are necessary for DNA binding. Proc Natl Acad Sci U S A 106:16604–16609. 19805344 10.1073/pnas.0908380106 PMC2745332

[27] Gallagher DT, Smith N, Kim SK, Robinson H, Reddy PT (2009) Profound asymmetry in the structure of the cAMP-free cAMP Receptor Protein (CRP) from Mycobacterium tuberculosis. J Biol Chem 284:8228–8232. 19193643 10.1074/jbc.C800215200 PMC2659179

[28] Lee TW, Won HS, Park SH, Kyogoku Y, Lee BJ (2001) Detection of the protein-protein interaction between cyclic AMP receptor protein and RNA polymerase, by (13)C-carbonyl NMR. J Biochem 130:57–61. 11432780 10.1093/oxfordjournals.jbchem.a002962

[29] Popovych N, Sun S, Ebright RH, Kalodimos CG (2006) Dynamically driven protein allostery. Nat Struct Mol Biol 13:831–838. 16906160 10.1038/nsmb1132 PMC2757644

[30] Tzeng SR, Kalodimos CG (2012) Protein activity regulation by conformational entropy. Nature 488:236–240. 22801505 10.1038/nature11271

[31] Toncrova H, McLeish TCB (2010) Substrate-Modulated Thermal Fluctuations Affect Long-Range Allosteric Signaling in Protein Homodimers: Exemplified in CAP. Biophys J 89:2317–2326. 10.1016/j.bpj.2010.01.039 PMC2872212 20483341

[32] Rodgers TL, Townsend PD, Burnell D, Jones ML, Richards SA, et al (2013) Modulation of Global Low-Frequency Motions Underlies Allosteric Regulation: Demonstration in CRP/FNR Family Transcription Factors. Plos Biology 11. 10.1371/journal.pbio.1001651 PMC3769225 24058293

[33] Rodgers TL, Burnell D, Townsend PD, Pohl E, Cann MJ, et al (2013) Delta Delta PT: a comprehensive toolbox for the analysis of protein motion. Bmc Bioinformatics 14. 10.1186/1471-2105-14-183 PMC3689072 23758746

[34] Bussmann M, Emer D, Hasenbein S, Degraf S, Eikmanns BJ, et al (2009) Transcriptional control of the succinate dehydrogenase operon sdhCAB of Corynebacterium glutamicum by the cAMP-dependent regulator GlxR and the LuxR-type regulator RamA. J Biotechnol 143:173–182. 19583988 10.1016/j.jbiotec.2009.06.025

[35] Panhorst M, Sorger-Herrmann U, Wendisch VF (2011) The pstSCAB operon for phosphate uptake is regulated by the global regulator GlxR in Corynebacterium glutamicum. Journal of Biotechnology 154:149–155. 20638427 10.1016/j.jbiotec.2010.07.015

[36] Studier FW (2005) Protein production by auto-induction in high density shaking cultures. Protein Expr Purif 41:207–234. 15915565 10.1016/j.pep.2005.01.016

[37] Letek M, Valbuena N, Ramos A, Ordonez E, Gil JA, et al (2006) Characterization and use of catabolite-repressed promoters from gluconate genes in Corynebacterium glutamicum. J Bacteriol 188:409–423. 16385030 10.1128/JB.188.2.409-423.2006 PMC1347311

[38] Teng T-Y (1990) Mounting of crystals for macromolecular crystallography in a free-standing thin film. J Appl Cryst 23:387–391.

[39] Kabsch W (2010) Xds. Acta Crystallogr D Biol Crystallogr 66:125–132. 20124692 10.1107/S0907444909047337 PMC2815665

[40] McCoy AJ (2007) Solving structures of protein complexes by molecular replacement with Phaser. Acta Crystallogr D Biol Crystallogr 63:32–41. 17164524 10.1107/S0907444906045975 PMC2483468

[41] Reddy MC, Palaninathan SK, Bruning JB, Thurman C, Smith D, et al (2009) Structural insights into the mechanism of the allosteric transitions of Mycobacterium tuberculosis cAMP receptor protein. J Biol Chem 284:36581–36591. 19740754 10.1074/jbc.M109.041343 PMC2794773

[42] Emsley P, Lohkamp B, Scott WG, Cowtan K (2010) Features and development of Coot. Acta Crystallogr D Biol Crystallogr 66:486–501. 20383002 10.1107/S0907444910007493 PMC2852313

[43] Murshudov GN, Skubak P, Lebedev AA, Pannu NS, Steiner RA, et al (2011) REFMAC5 for the refinement of macromolecular crystal structures. Acta Crystallogr D Biol Crystallogr 67:355–367. 21460454 10.1107/S0907444911001314 PMC3069751

[44] Laskowski RA, Macarthur MW, Moss DS, Thornton JM (1993) Procheck - a Program to Check the Stereochemical Quality of Protein Structures. Journal of Applied Crystallography 26:283–291.

[45] Chu SY, Tordova M, Gilliland GL, Gorshkova I, Shi Y, et al (2001) The structure of the T127L/S128A mutant of cAMP receptor protein facilitates promoter site binding. J Biol Chem 276:11230–11236. 11124966 10.1074/jbc.M010428200

[46] Mosca R, Schneider TR (2008) RAPIDO: a web server for the alignment of protein structures in the presence of conformational changes. Nucleic Acids Res 36:W42–46. 18460546 10.1093/nar/gkn197 PMC2447786

[47] Tzeng SR, Kalodimos CG (2009) Dynamic activation of an allosteric regulatory protein. Nature 462:368–372. 19924217 10.1038/nature08560

[48] Moore JL, Gorshkova II, Brown JW, McKenney KH, Schwarz FP (1996) Effect of cAMP binding site mutations on the interaction of cAMP receptor protein with cyclic nucleoside monophosphate ligands and DNA. J Biol Chem 271:21273–21278. 8702903 10.1074/jbc.271.35.21273

[49] Stapleton M, Haq I, Hunt DM, Arnvig KB, Artymiuk PJ, et al (2010) Mycobacterium tuberculosis cAMP receptor protein (Rv3676) differs from the Escherichia coli paradigm in its cAMP binding and DNA binding properties and transcription activation properties. J Biol Chem 285:7016–7027. 20028978 10.1074/jbc.M109.047720 PMC2844151

[50] Passner JM, Schultz SC, Steitz TA (2000) Modeling the cAMP-induced allosteric transition using the crystal structure of CAP-cAMP at 2.1 A resolution. J Mol Biol 304:847–859. 11124031 10.1006/jmbi.2000.4231

[51] Yu S, Lee JC (2004) Role of residue 138 in the interdomain hinge region in transmitting allosteric signals for DNA binding in Escherichia coli cAMP receptor protein. Biochemistry 43:4662–4669. 15096034 10.1021/bi0362166

[52] Won HS, Lee YS, Lee SH, Lee BJ (2009) Structural overview on the allosteric activation of cyclic AMP receptor protein. Biochim Biophys Acta 1794:1299–1308. 19439203 10.1016/j.bbapap.2009.04.015

[53] Kumar P, Joshi DC, Akif M, Akhter Y, Hasnain SE, et al (2010) Mapping conformational transitions in cyclic AMP receptor protein: crystal structure and normal-mode analysis of Mycobacterium tuberculosis apo-cAMP receptor protein. Biophys J 98:305–314. 20338852 10.1016/j.bpj.2009.10.016 PMC2808490

[54] Pohl E, Holmes RK, Hol WG (1998) Motion of the DNA-binding domain with respect to the core of the diphtheria toxin repressor (DtxR) revealed in the crystal structures of apo- and holo-DtxR. J Biol Chem 273:22420–22427. 9712865 10.1074/jbc.273.35.22420

[55] Hong M, Fuangthong M, Helmann JD, Brennan RG (2005) Structure of an OhrR-ohrA operator complex reveals the DNA binding mechanism of the MarR family. Mol Cell 20:131–141. 16209951 10.1016/j.molcel.2005.09.013

[56] Frenois F, Engohang-Ndong J, Locht C, Baulard AR, Villeret V (2004) Structure of EthR in a ligand bound conformation reveals therapeutic perspectives against tuberculosis. Mol Cell 16:301–307. 15494316 10.1016/j.molcel.2004.09.020

[57] Thompson JD, Higgins DG, Gibson TJ (1994) CLUSTAL W: improving the sensitivity of progressive multiple sequence alignment through sequence weighting, position-specific gap penalties and weight matrix choice. Nucleic Acids Res 22:4673–4680. 7984417 10.1093/nar/22.22.4673 PMC308517

[58] Gouet P, Courcelle E, Stuart DI, Metoz F (1999) ESPript: analysis of multiple sequence alignments in PostScript. Bioinformatics 15:305–308. 10320398 10.1093/bioinformatics/15.4.305

[59] Brunger AT (1992) Free R value: a novel statistical quantity for assessing the accuracy of crystal structures. Nature 355:472–475. 18481394 10.1038/355472a0

